# Unraveling the Genetic Architecture of Obesity: A Path to Personalized Medicine

**DOI:** 10.3390/diagnostics15121482

**Published:** 2025-06-11

**Authors:** Faisal Kunnathodi, Amr A. Arafat, Waleed Alhazzani, Mohammad Mustafa, Sarfuddin Azmi, Ishtiaque Ahmad, Jamala Saleh Selan, Riyasdeen Anvarbatcha, Haifa F. Alotaibi

**Affiliations:** 1 Health Research Center, Ministry of Defense Health Services, Riyadh 12485, Saudi Arabia; amr.arafat@med.tanta.edu.eg (A.A.A.); waleed.al-hazzani@medportal.ca (W.A.); khan.mdmustafa@gmail.com (M.M.); sarfu_azmi@yahoo.co.in (S.A.); ishatiaquehamdard@gmail.com (I.A.); ksu.jss@gmail.com (J.S.S.); anfariyas@gmail.com (R.A.); halotaibe@mod.gov.sa (H.F.A.); 2 Departments of Adult Cardiac Surgery, Prince Sultan Cardiac Center, Riyadh 31982, Saudi Arabia; 3 Critical Care and Internal Medicine Department, College of Medicine, Imam Abdulrahman Bin Faisal University, Dammam 31441, Saudi Arabia; 4 Department of Family Medicine, Prince Sultan Military Medical City, Riyadh 11159, Saudi Arabia

**Keywords:** obesity treatment, personalized medicine, genomics, genome-wide association studies (GWAS), epigenetics

## Abstract

Obesity is a global health challenge characterized by significant heterogeneity in causes and treatment responses, complicating sustainable management. This narrative review explores the genomic architecture of obesity and its implications for personalized interventions, focusing on how genetic variations influence key biological pathways and treatment outcomes. A comprehensive literature search, guided by the authors’ expertise, was conducted to identify key publications on the genomics of obesity and personalized approaches. The selection of articles prioritized those that provided direct insights into the genomic basis of obesity and its potential for informing tailored strategies. Genomic studies reveal both monogenic and polygenic influences on obesity, identifying numerous susceptibility loci. Genome-wide association studies (GWASs) have linked common variants in genes like *FTO* and *MC4R* to increased BMI and appetite dysregulation, respectively. Epigenetic research highlights the role of DNA methylation and other modifications in gene–environment interactions. Genetic and polygenic risk scores (GRSs and PRSs) show potential for refining risk stratification and predicting treatment response. The gut microbiome and metabolome also contribute to obesity pathogenesis, offering novel targets for intervention. Personalized medicine offers significant potential for improving obesity management through tailored interventions based on an individual’s genetic and ‘omics’ profile. Future research should focus on elucidating the functional consequences of identified variants, exploring gene–environment interactions, and developing strategies to overcome current limitations in clinical translation. With continued advancements, precision medicine can enhance treatment efficacy, increase sustainability, and help reduce the global burden of obesity-related diseases.

## 1. Introduction

Obesity, a global health crisis of escalating proportions, affects over 890 million adults worldwide, posing a significant threat to public health and economic stability [1,2]. Beyond the well-established associations with increased risk of type 2 diabetes, cardiovascular disease, and certain cancers, obesity presents a unique challenge due to its marked heterogeneity [3,4,5,6,7,8]. While readily modifiable environmental factors, such as diet and physical activity, undeniably contribute to the development of obesity, the intricate interplay of genetics, epigenetics, the gut microbiome, and other ‘omics’ factors ultimately dictates individual susceptibility and response to conventional interventions [9,10,11,12,13]. This inherent variability highlights the limitations of traditional ‘one-size-fits-all’ approaches and underscores the urgent need for personalized strategies that account for individual differences.

**Figure 1:**
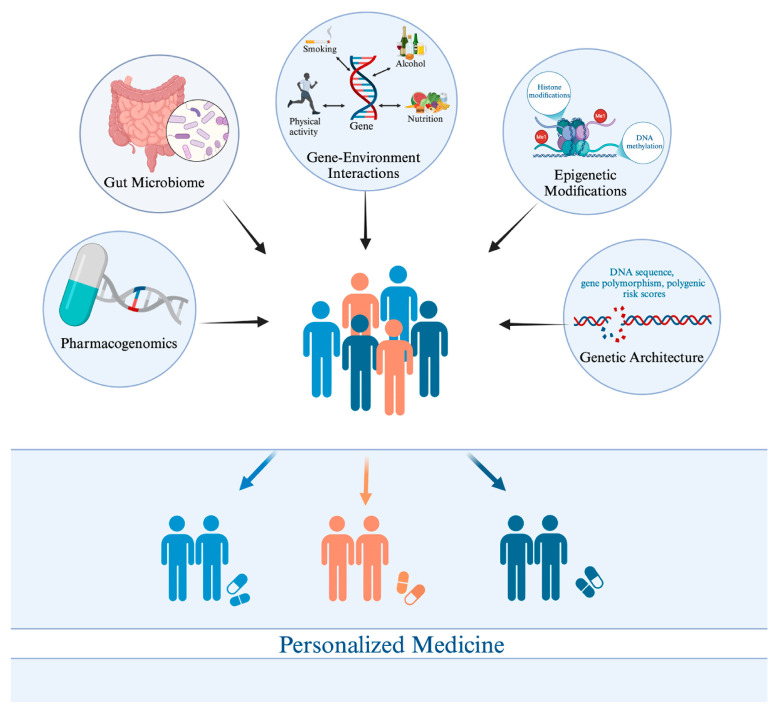
The genomic landscape of personalized obesity management.

Precision medicine, defined as an evolving approach to healthcare that tailors interventions to the unique characteristics of each individual, taking into account their genes, environment, and lifestyle, offers a promising framework for addressing obesity’s complexities [14,15,16,17]. Unlike traditional approaches that rely on population-based averages, precision medicine seeks to identify and target the underlying mechanisms driving obesity in each individual. Advances in genomics have been instrumental in this effort, leading to the discovery of numerous susceptibility loci and genetic variants that influence key processes, such as appetite regulation, energy metabolism, and adipogenesis [18,19,20,21,22,23,24,25]. These discoveries, coupled with burgeoning insights into epigenetic modifications, the composition and function of the gut microbiome, and circulating metabolomic signatures, are collectively paving the way for targeted interventions that address the specific biological drivers of obesity in each patient. Figure 1 illustrates the genomic insights driving personalized medicine in obesity treatment.

This review aims to synthesize current knowledge on the genomic architecture of obesity and explore its far-reaching implications for the development of personalized interventions. We will examine how genomic and other ‘omics’ data can be leveraged to (1) refine risk stratification and identify individuals most likely to benefit from early intervention; (2) predict individual treatment response to diet, exercise, and pharmacotherapy; and (3) develop tailored strategies that enhance efficacy, minimize adverse effects, and promote sustainable outcomes in the long-term management of obesity. By highlighting the rapidly evolving landscape of genomic insights and their translational potential, this review seeks to inform future research directions and accelerate the integration of personalized medicine approaches into routine clinical practice, ultimately improving the lives of individuals affected by this complex and multifaceted disease.

## 2. Materials and Methods

This narrative review synthesizes the current literature on the genomic architecture of obesity and its implications for personalized interventions. Drawing upon the authors’ expertise in the field, a comprehensive literature search was conducted to identify key publications relevant to these topics. Search terms included ‘personalized medicine’, ‘genomics’, ‘epigenetics’, ‘multi-omics approaches’, and ‘obesity treatment’. The selection of articles prioritized those that provided direct insights into the genomic basis of obesity and its potential for informing personalized strategies. This approach resulted in the inclusion of 111 articles that were deemed most pertinent to the scope and objectives of this review. The review examines the influence of genetic architecture, GWAS, epigenome-wide association studies (GWASs), nutrigenetics, pharmacogenomics, the gut microbiome, and metabolomic signatures on obesity treatment outcomes, while also considering the challenges of translating these findings into clinical practice.

## 3. Genetic Architecture

Extensive research, including twin, familial, and adoption studies, has established a significant genetic predisposition to obesity [11,26]. These studies suggest that the heritability of obesity ranges from 40 to 77%, indicating that genetic factors play a crucial role [27]. Interestingly, a predisposition toward being slim also appears to be inherent [28]. Obesity can be broadly categorized based on its genetic architecture as polygenic and monogenic, with the vast majority of cases being polygenic [29].

**Figure 2:**
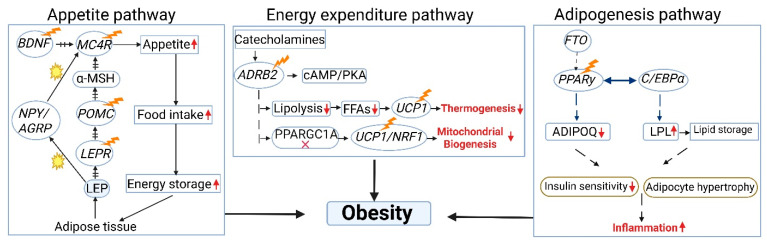
Schematic representation of key molecular pathways in appetite regulation, energy expenditure, and adipogenesis relevant to obesity’s pathophysiology. Appetite regulation: *BDNF* activates *MC4R* via *POMC*-derived α-MSH, while *NPY*/*AGRP* inhibit *POMC*, promoting food intake. *LEPR* signaling suppresses this drive. Mutations in *LEPR*, *POMC*, or *BDNF* impair *MC4R* activation, leading to hyperphagia. Energy expenditure: Catecholamines activate *ADRB2*, triggering cAMP/PKA signaling, lipolysis, and *UCP1*-mediated thermogenesis. PPARGC1A supports mitochondrial biogenesis. Disruption reduces energy expenditure. Adipogenesis: *PPARγ* and *C*/*EBPα* regulate adipocyte differentiation via *ADIPOQ* and *LPL*. Mutations in *PPARγ* reduce adiponectin, increase lipid storage, and promote inflammation and insulin resistance. Symbols denote genetic mutations (lightning bolt), activation (energy symbol), increased or decreased activity (upward/downward arrows), inhibition (cross mark), modulation (dotted arrow), and inactivation or blockade (arrow with block lines). (FFAs, Free Fatty Acids; UCP, Uncoupling Proteins; PPARGC1A, Peroxisome Proliferator-Activated Receptor Gamma Coactivator 1-Alpha; *POMC*, proopiomelanocortin; *LEPR*, leptin receptor; *BDNF*, Brain-Derived Neurotrophic Factor; *MC4R*, Melanocortin 4 Receptor; *NPY*/*AGRP*, Neuropeptide Y/Agouti-Related Protein; *ADRB2*, Beta-2 Adrenergic Receptor; *NRF*, Nuclear Respiratory Factors; *FPARγ*, Peroxisome Proliferator-Activated Receptor Gamma; *ADIPOQ*, adiponectin; *LPL*, Lipoprotein Lipase).

Polygenic obesity, often referred to as common obesity and accounting for the vast majority of cases, is multifactorial, resulting from the cumulative effects of numerous common gene variants (single-nucleotide polymorphisms or SNPs) in combination with lifestyle factors [29]. In contrast, monogenic obesity, caused by a single, rare mutation in genes like *LEP*, *LEPR*, *POMC*, or *MC4R*, accounts for less than 1% of obesity cases. Regardless of the specific genetic architecture, these genetic predispositions influence various physiological processes, including appetite and satiety (e.g., variations in *BDNF*, *MC4R*, *NEGR*) [18,19,20], energy and lipid metabolism (e.g., *FTO*, *RPTOR*, *MAP2K5*) [21,22,23], insulin secretion and action (e.g., *TCF7L2*, *IRS1*) [21], and adipogenesis [21]. While individually these variants have small effects, their combined impact can significantly increase an individual’s susceptibility to weight gain. Understanding an individual’s polygenic risk score (PGS), based on the cumulative effect of these common variants, may help to personalize dietary and lifestyle recommendations for more effective weight management. As summarized in Table 1, monogenic and polygenic obesity differ in their genetic origins, clinical presentation, and potential for targeted interventions, each offering unique insights for personalized care [13]. To further illustrate the biological underpinnings of obesity, Figure 2 highlights how genetic disruptions in key molecular pathways, such as the melanocortin system, adrenergic signaling, and adipocyte differentiation, can influence appetite, energy expenditure, and lipid storage, ultimately contributing to obesity development. Together, the genetic distinctions outlined in Table 1 and the mechanistic pathways illustrated in Figure 2 underscore the multifactorial nature of obesity and the potential for genomics to inform individualized treatment strategies.

Gene discovery efforts have revealed that the genetic and metabolic foundations of both polygenic and monogenic obesity are interconnected, emphasizing the critical role of the brain in regulating body weight [21]. This insight confirms the complexity of obesity and underscores the importance of understanding genetic factors to develop effective interventions.

## 4. Genomic and Epigenomic Landscapes of Obesity

Genome-wide association studies (GWAS) have revolutionized our understanding of the genetic architecture of obesity. By leveraging large datasets and high-throughput genotyping technologies, GWAS have identified thousands of genetic loci associated with BMI and other adiposity measures [30]. These studies have revealed that obesity is a highly polygenic trait, with numerous common variants exerting small, cumulative effects on an individual’s susceptibility [21]. Since the initial discovery of *FTO* variants in 2007 [22], meta-analyses of large-scale GWAS have uncovered over 1000 loci, solidifying the understanding of common obesity [21]. These studies have also revealed that many obesity-related genes are linked to other chronic metabolic conditions, such as type 2 diabetes and cardiovascular disease, often sharing common metabolic pathways [31]. This overlap suggests a shared genetic etiology and potential for targeted interventions that address multiple aspects of metabolic health. While the majority of GWAS have been conducted in individuals of European descent, it is important to note that robust investigations in Asian, African, and Hispanic populations have identified novel and population-specific susceptibility loci, expanding the diversity of our knowledge and highlighting the importance of considering ancestry in genetic studies [23,32,33,34,35]. For instance, studies in the Greenlandic population have identified obesity-related polymorphisms in the *ADCY3* gene [36,37], which may influence body weight control through mechanisms distinct from those identified in European populations [38]. Despite these advances, it is crucial to acknowledge that GWAS findings collectively explain only a small fraction (approximately 6%) of the variation in BMI [39], underscoring the complexity of obesity and the importance of considering non-genetic factors. It is also worth noting the intriguing finding that the predominant expression of obesity-associated genes occurs in the central nervous system, rather than peripheral tissues, like adipose tissue [21]. This observation highlights the critical role of hypothalamic circuits in regulating energy balance and suggests that targeting these neural pathways may be a promising avenue for therapeutic intervention. Furthermore, research has indicated that a substantial genetic component influences eating behaviors and dietary preferences, with 83 out of 85 food behaviors showing evidence of heritability and association with various SNPs [40].

Epigenome-wide association studies (EWAS) complement GWAS by exploring the role of epigenetic mechanisms, such as DNA methylation and histone modifications, in regulating gene expression and influencing obesity susceptibility [41,42]. Unlike GWAS, which focus on inherited genetic variations, EWAS investigate how environmental factors can alter gene expression through epigenetic modifications. These epigenetic modifications are thought to regulate gene–environment interactions [41,42]. EWAS have revealed correlations between DNA methylation patterns in genes related to eating behaviors, lipid and glucose metabolism, and adipogenesis and obesity-related traits and comorbidities [24]. These epigenetic modifications are influenced by environmental factors, such as nutritional intake, physical activity, and surgical interventions, underscoring the dynamic interplay between genes and the environment [43,44,45]. Similarly to GWAS, EWAS have identified specific genes, including *CPT1A*, *PGC1A*, *ABCG1*, and *HIF3A*, that show correlations between BMI and altered methylation sites [46,47,48,49,50,51,52,53,54]. For instance, studies have shown that methylation patterns in *CPT1A*, a gene involved in fatty acid metabolism, are associated with both BMI and altered metabolic health [46,47,55]. Moreover, genetic variations can also influence methylation patterns at specific CpG sites, leading to co-methylation and subsequent changes in clinical features, suggesting a link between genotype and phenotype [56].

While both GWAS and EWAS have identified numerous candidate genes and epigenetic markers associated with obesity, translating these findings into clinically actionable insights remains a challenge. For example, the *FTO* locus, initially implicated in obesity risk by GWAS [22], has been extensively studied, with research elucidating the regulatory mechanisms involving *ARID5B* and its effects on *IRX3* and *IRX5* expression [57]. Engaging in physical activity can reduce the effect of *FTO* SNPs on obesity susceptibility by approximately 30% [58], and evidence suggests that *FTO* influences food preferences and intake [59]. However, the precise mechanisms through which many other GWAS and EWAS signals influence obesity development remain unclear, necessitating further functional studies. Moreover, despite the potential of epigenetic data as predictive and diagnostic tools, their clinical application in personalized obesity management is currently limited. This highlights the need for ongoing research and development in this field to increase the clinical utility of epigenetic data.

A comprehensive overview of key genetic loci identified through GWAS and epigenetic markers revealed through EWAS is presented in Table 2 and Table 3, respectively, highlighting the multifaceted genomic and epigenomic contributions to obesity across diverse populations.

Ultimately, integrating genomic and epigenomic data holds promise for refining our understanding of obesity’s etiology and developing more effective, personalized interventions. For instance, by combining an individual’s genetic risk score with their methylation profile, it may be possible to predict their response to specific dietary interventions or pharmacological agents. Future research should focus on elucidating the functional consequences of identified genetic variants and epigenetic marks, exploring gene–environment interactions, and developing novel strategies to translate these findings into clinical practice.

## 5. Genetic and Polygenic Risk Scores for Obesity

In light of the advancements in understanding the genetic architecture of obesity, genetic risk scores (GRSs) and polygenic risk scores (PRSs) have emerged as tools for assessing an individual’s overall genetic predisposition to the disease. GRSs are typically constructed by summing the number of risk alleles an individual carries across a selected panel of genetic variants associated with obesity, often weighted by their effect size [95]. PRSs, on the other hand, represent a more comprehensive approach, encompassing potentially thousands or even millions of common genetic variants identified through GWASs [96]. These variants are weighted by their effect sizes estimated from large-scale GWAS and aggregated into a single, quantitative measure of genetic susceptibility. For example, a PRS developed by Khera et al. [96] demonstrated the ability to categorize individuals of European descent according to their likelihood of developing obesity. This PRS not only predicted disparities in body weight trajectories but also the chance of developing severe obesity in adolescence, even in individuals with only minor differences in body weight at birth. More recently, researchers have also begun to develop scores for methylation risk, utilizing data from EWAS to identify methylated CpG sites associated with obesity [86].

While GRSs and PRSs hold promise for identifying individuals at high risk of obesity and predicting their response to interventions, it is important to acknowledge their current limitations. Despite their ability to capture a significant portion of the heritable risk, these scores often fail to provide precise or robust predictions at the individual level [97]. Moreover, the predictive power of GRSs and PRSs can be influenced by various factors, including ancestry, sex, and environmental exposures. For instance, studies have shown that factors like low socioeconomic status, chronic psychological stress, reduced sleep duration, high consumption of sugary beverages and fried foods, and lack of physical activity can amplify the relationship between genetic risk scores and BMI [97].

Despite these limitations, GRSs and PRSs have the potential to contribute to personalized approaches to obesity management. By identifying individuals at increased genetic risk, these scores may help to target more intensive and personalized preventative interventions relating to diet and exercise, such as lifestyle counseling and dietary modifications, for those most likely to benefit. Furthermore, GRSs and PRSs could potentially be used to stratify individuals in clinical trials, allowing researchers to assess the efficacy of different interventions, like pharmacotherapy or tailored exercise programs, in genetically defined subgroups. Future research should focus on improving the accuracy and predictive power of GRSs and PRSs, exploring their interactions with environmental factors, and evaluating their clinical utility in diverse populations.

## 6. Nutrigenetics and Obesity: Impact on Underlying Pathologies

Nutrition undeniably plays a key role as an environmental factor that interacts with an individual’s genetic makeup, influencing their susceptibility to obesity and related metabolic disorders [98]. However, the field of nutrigenetics moves beyond simply acknowledging this interaction by exploring the specific mechanisms through which genetic variations influence an individual’s response to nutrients and dietary patterns, ultimately impacting underlying pathologies. One key area of investigation is how genetic variations affect lipid metabolism and inflammation. For instance, individual responses to omega-3 fatty acids, known for their anti-inflammatory properties, can vary significantly based on genetic polymorphisms [99,100]. Research has indicated that genetic variations in genes influencing adipogenesis and inflammation, such as *PPARG* or *FADS1*/*FADS2*, may influence the extent to which omega-3 supplementation affects body composition, waist circumference, or adipokine levels. Individuals with certain *PPARG* variants, for example, may exhibit reduced benefits from omega-3 supplementation in terms of reducing inflammation and improving insulin sensitivity, highlighting the need for personalized recommendations [99,100]. Another area of focus is the impact of genetic variations on carbohydrate metabolism and insulin sensitivity. Variations in genes involved in glucose transport, insulin signaling, or glycogen synthesis can influence an individual’s response to different carbohydrate sources and dietary glycemic loads. For example, individuals with certain variants in the *TCF7L2* gene, which is associated with type 2 diabetes risk, may benefit from diets with a lower glycemic index to improve insulin sensitivity and reduce the risk of weight gain [101]. Furthermore, nutrigenetics research is exploring how genetic variations influence appetite regulation and energy expenditure. Variations in genes involved in taste perception, reward pathways, or thermogenesis can affect an individual’s food preferences, satiety signals, and metabolic rate. For instance, individuals with certain variants in the *TAS2R38* gene, which influences the perception of bitter taste, may be more likely to consume sugary beverages, potentially increasing their risk of weight gain [102].

While the field of nutrigenetics holds great promise for personalized nutrition, several challenges remain. The acceptability, privacy protection, marketing delivery and distribution, affordability, cost, and dependability of personalized nutrition for obesity implementation are still subject to debate. In many nations, direct-to-consumer genetic testing is already being utilized to provide customized medicines based on genotype, and it is becoming increasingly helpful in managing obesity [103]. However, there are ongoing concerns regarding the consistency and interpretation of genetic risk assessments, as well as public attitudes toward using these tests for estimating lifetime disease risk [104].

Ultimately, further research is needed to evaluate the use, effectiveness, and impact of genome-wide profiling in disease risk assessment and treatment guidance. Methodological and ethical concerns must also be taken into consideration [104]. In light of these challenges, alternative approaches to implementing personalized dietary strategies should be considered. For example, consultations with experts who use genetic information to assess obesity risk and progression could be an effective way to manage or prevent obesity. There is conflicting evidence about the potential benefits of this method over standard medical care for weight loss [105,106].

## 7. The Gut Microbiome and Metabolome in Obesity: Implications for Metabolic Dysfunction and Personalized Approaches

The gut microbiome, a complex community of microorganisms residing in the digestive tract, and the host metabolome, the collection of small molecules produced by both the host and the microbiome, have emerged as critical players in the pathogenesis of obesity and related metabolic disorders [107]. The composition and function of the gut microbiome are influenced by a variety of factors, including genetics, diet, and environmental exposures, highlighting the intricate interplay between host and microbe [107]. The gut microbiome contributes to obesity through multiple mechanisms, including energy extraction from food, appetite regulation, inflammation, and the modulation of host metabolic pathways [108]. Specific alterations in the gut microbiome’s composition have been linked to obesity, including decreased microbial diversity, reduced abundance of beneficial bacteria, such as *Akkermansia muciniphila* and *Faecalibacterium prausnitzii*, and an increased Firmicutes to Bacteroidetes ratio [109,110]. Building on these findings, next-generation probiotics, such as *A. muciniphila*, *F. prausnitzii*, and *Eubacterium hallii*, have shown promise due to their beneficial roles in host metabolism. *A. muciniphila* is associated with improved gut barrier integrity and reduced metabolic endotoxemia, while *F. prausnitzii* exerts anti-inflammatory effects through butyrate production. *E. hallii* contributes to energy homeostasis by producing short-chain fatty acids (SCFAs), such as butyrate and propionate, which regulate glucose and lipid metabolism [110]. These findings support the potential of targeted microbiome modulation as a personalized therapeutic strategy in obesity management.

The gut microbiome also plays a critical role in the pathogenesis of metabolic syndrome, a cluster of conditions including obesity, insulin resistance, dyslipidemia, and hypertension [111]. Dysbiosis, or alterations in the gut microbiome’s composition and function, has been shown to promote metabolic endotoxemia, increase hepatic lipogenesis, and impair insulin signaling, contributing to the development of metabolic syndrome [111]. Furthermore, the microbiome influences SCFA production, including acetate, propionate, and butyrate, which regulate appetite, glucose homeostasis, and immune function [112].

In addition to the gut microbiome, metabolomics offers valuable insights into the metabolic signatures associated with obesity. An aberrant metabolome, defined by elevated levels of branched-chain amino acids (BCAAs), altered lipid profiles, and disrupted energy metabolism, has been observed not only in individuals with obesity but also in metabolically unhealthy lean individuals and even in some apparently healthy populations. These metabolic alterations are associated with an increased risk of cardiovascular disease and other metabolic complications [113]. Furthermore, a person’s initial metabolic profile can be used to predict their response to weight loss interventions, highlighting the potential for personalized approaches [114,115].

Emerging evidence suggests that microRNAs (miRNAs), small non-coding RNA molecules that regulate gene expression, may also play a role in the pathogenesis of obesity and related metabolic disorders [116]. MicroRNAs can be secreted by various cell types, including immune cells and adipocytes, and they can influence gene expression in target cells, affecting processes like inflammation, insulin signaling, and lipid metabolism. Specific miRNAs have been identified as potential biomarkers for obesity and metabolic syndrome, offering new avenues for diagnosis and prevention [116].

The integration of gut microbiome and metabolomic data holds great promise for developing personalized approaches to managing obesity and improving metabolic health. By characterizing an individual’s gut microbiome composition and metabolomic profile, it may be possible to identify specific metabolic imbalances and tailor interventions to address these imbalances. For example, dietary interventions, such as prebiotics and probiotics, can be used to modulate the gut microbiome’s composition and function, promoting the growth of beneficial bacteria and reducing inflammation [117,118]. Furthermore, personalized dietary recommendations based on an individual’s gut microbiome and metabolomic profile may enhance the effectiveness of weight loss interventions and improve long-term metabolic outcomes [119]. Future research should focus on elucidating the complex interactions between the microbiome, the metabolome, and the host genome to develop novel, individualized strategies for obesity treatment.

## 8. Pharmacotherapy for Monogenic Obesity Disorders: A Model for Personalized Targeting

While monogenic obesity accounts for a small percentage of cases, it provides a powerful model for understanding how specific genetic defects can be targeted with personalized therapies [120]. Currently, there are no established clinical treatments specifically targeting these mutations, and most of these cases are of polygenic origin. Leptin receptor and proopiomelanocortin deficiency are monogenic obesity disorders caused by bi-allelic variants in *LEPR* and *POMC* or *PCSK1*, respectively [121]. These illnesses are underdiagnosed because they are uncommon and typically overlooked by healthcare professionals when a differential diagnosis for obesity is made [109]. However, patients with these monogenic mutations have demonstrated the potential of precision medicine to impact clinical outcomes significantly, a theme that will be explored in detail throughout this text.

For example, obesity related to congenital leptin deficiency has been successfully treated with recombinant leptin [122]. This approach directly replaces the missing protein, restoring normal appetite regulation and energy expenditure. Likewise, individuals deficient in *POMC* or *LEPR* have shown encouraging results in terms of weight loss while using setmelanotide, an agonist of the MC4R, which is the receptor through which *POMC* signals [121,122,123,124]. The Food and Drug Administration has approved setmelanotide for homozygous mutations in *PSCK1*, *LEPR*, and *POMC*. These genes are some of the few involved in *POMC* signaling. It has been demonstrated that using setmelanotide for these mutations reduces appetite and body weight, although the effects are inconsistent [125]. The fact that some individuals with common obesity also respond to these treatments suggests the presence of SNPs that share a similar molecular mechanism of action [123,126]. Identifying these SNPs could offer additional therapeutic options for a subset of individuals with common obesity who might benefit from these medications.

These examples highlight the power of personalized medicine to target the root cause of disease in individuals with specific genetic defects. This approach underscores the importance of accurate diagnosis and genetic testing to identify appropriate candidates for targeted therapies. Furthermore, the success of these targeted therapies provides a proof of concept for developing similar strategies for other, more common forms of obesity driven by specific genetic or metabolic abnormalities.

## 9. Challenges in Personalized Medicine for Obesity

The implementation of precision medicine in clinical practice faces several challenges, particularly due to the complex and multifaceted nature of obesity. One major challenge is addressing the heterogeneity of obesity and developing tailored strategies that target the specific factors affecting each individual’s condition. Furthermore, our understanding of the genetic basis of obesity is incomplete. Only a small percentage of the total obesity risk is caused by the numerous known genetic variations linked to obesity, and their effect sizes are small. The link between genetics and obesity is further complicated by gene–environment interactions and epigenetic variables. The application of personalized medicine in the treatment of obesity necessitates the integration of many omics data types, such as genomics, transcriptomics, metabolomics, and microbiomics, to create comprehensive individual profiles and inform treatment choices. Large-scale omics datasets present computational and technological difficulties regarding integration and analysis. Translating personalized medicine techniques from experimental settings into standard clinical practice presents considerable practical and logistical obstacles. Healthcare professionals need tools and training to analyze genetic and omics data properly, incorporate tailored therapies into clinical processes, and explain complicated medical information to patients in a way they can comprehend. The application of personalized medicine in the treatment of obesity needs to consider how behavioral and psychosocial aspects influence people’s food preferences, levels of physical activity, and compliance with treatment plans. To address these hurdles, interdisciplinary cooperation among researchers, medical professionals, legislators, and patients is necessary to create evidence-based, customized medicine strategies that increase the efficiency, availability, and long-term viability of obesity therapy.

## 10. Conclusions

Personalized medicine is revolutionizing obesity management by unraveling the complex interplay of genetics and environmental factors. Genomic and epigenomic insights are guiding the development of tailored interventions, while tools like GRSs and PRSs enable customized risk assessments. Despite these advancements, challenges remain in integrating multi-omics data and addressing translational barriers. By leveraging genomic insights and fostering collaborative efforts, we can pave the way for more precise and sustainable solutions to combat the obesity epidemic, ultimately improving health outcomes worldwide. Future research should focus on elucidating the functional consequences of identified genetic variants, exploring gene–environment interactions, and developing novel strategies to translate these findings into clinical practice.

## Figures and Tables

**Table 1:** Key differences between monogenic and polygenic obesity.

Feature	Monogenic Obesity	Polygenic Obesity
Genetic Cause	Single, highly penetrant mutation in a key gene	Cumulative effect of multiple common variants with small individual effects
Example Genes	*LEP*, *LEPR*, *POMC*, *MC4R*	*FTO*, *TCF7L2*, *IRS1*
Typical Onset	Severe, early-onset (often in infancy or early childhood)	Variable, can present at any age, but often develops in adulthood
Prevalence	Rare (<1% of obesity cases)	Common (>95% of obesity cases)
Effect on Specific Pathways	Disrupts a key regulatory pathway (e.g., leptin signaling)	Influences multiple pathways (appetite, metabolism, adipogenesis)
Environmental Influence	Less susceptible to environmental factors	More susceptible to environmental fators
Potential for Targeted Therapy	High (e.g., leptin replacement, MC4R agonists)	Emerging (e.g., personalized dietary recommendations based on GRS, PRS)

**Table 2:** Summary of genome-wide association studies (GWAS) identifying genetic variants associated with obesity-related traits.

Study ID	Study Population	Key Gene(s)/Locus (Selected)	Key Findings
Akiyama et al., 2017 [23]	Japanese	*GPR101*, *GPR75*, *KCNQ1*	Associated with increased BMI
Ang et al., 2023 [60]	Primarily European ancestry	*MTOR*, *MAP2K5*, *RBFOX1*, *EP300*, *DNM1*	Associated with increased obesity risk
Chehadeh et al., 2020 [35]	Young Emirati adults	*FTO*, *MC4R, TMEM18*	*FTO* variant rs3751812 linked to obesity in males, while MC4R and TMEM18 associated with females
Chiang et al., 2019 [33]	Han Chinese	*FTO*	Associated with morbid obesity
Chung et al., 2022 [61]	European ancestry	*APOE*	C allele of rs429358 increasingly associated with lower body fat percentage with age
Felix et al., 2016 [62]	European ancestry	*ELP3*, *RAB27B*, *ADAM23*	Associated with increased adiposity in childhood
Hägg et al., 2015 [63]	European ancestry	*TXNDC12*, *PEX2*, *SSFA2*	Associated with increased adiposity
Heid et al., 2010 [64]	European ancestry	*RSPO3*, *VEGFA*, *GRB14*, *LYPLAL1*, *ITPR2*, *HOXC13*, *ADAMTS*	Associated with increased WHR
Hinney et al., 2007 [65]	German ancestry	*FTO*	rs1121980 associated with increased obesity risk
Kilpeläinen et al., 2011 [66]	European ancestry	*LEP*, *SLC32A1*, *GCKR*, *CCNL1*, *FTO*	Associated with increased adiposity and increased leptin
Lee, 2022 [67]	European population	*INPP4B*, *CHRNB4*	Associated with decreased BMI in smokers
Li et al., 2010 [68]	European descent	*FTO*, *TMEM18*, *MC4R*, *FAIM2*	Variants in these genes showed associations with increased BMI and obesity
Liu et al., 2008 [69]	U.S. and French Caucasian populations	*CTNNBL1*	rs6013029 particularly associated with increased BMI and fat mass
Liu et al., 2016 [70]	African American adults	*NEGR1*, *NRXN3*, *BDNF*, *ADCY3*, *FTO*	Multi-variant interactions involving these genes were associated with obesity risk
Liu et al., 2018 [71]	European and non-European ancestry	*TUFM*, *SPI*, *APOBR*, *CPEB4*	*TUFM*, *SPI*, *APOBR*, and *CPEB4* associated with BMI and WHR, respectively
Locke et al., 2015 [21]	European ancestry	*FTO*	Associated with increased BMI
Lv et al., 2015 [72]	Chinese children and adolescents	*MC4R*, *SEC16B*, *MAP2K5*, *KCTD15*	Associated with increased obesity risk
Mägi et al., 2013 [73]	European adults	*FTO*, *NEGR1*	*FTO* (rs9939609) and *NEGR1* (rs2815752) loci associated with obesity
Mejía-Benítez et al., 2013 [74]	Mexican children	*GNPDA2*	*GNPDA2* (rs10938397) locus associated with BMI
Pei et al., 2014 [75]	Diverse ethnic backgrounds	*CTSS*, *NLK*, *FTO*, *MC4R*, *TMEM18*	*CTSS* and *NLK* were linked to lower fat mass; *FTO* and *MC4R* to higher BMI; *TMEM18* to lower BMI
Pulit et al., 2019 [76]	European ancestry	*FTO*	Associated with increased BMI and WHR
Rask-Andersen et al., 2012 [77]	Swedish and Greek children	*AP2K5*	*AP2K5*-linked SNP rs2241423 associated with lower BMI
Shungin et al., 2015 [78]	Mainly European ancestry	*LEKR1*, *CCDC92*, *VEGFA*, *RSPO3*	Positively associated with body fat distribution
Takahashi et al., 2024 [34]	Japanese adults	*FTO*, *MC4R*, *SEC16B*, *BDNF*	*FTO*, *MC4R*, and *SEC16B* positively associated with higher BMI, while *BDNF* showed negative associations
Tang et al., 2024 [79]	European ancestry	*US*, *STX4*, *CCNT2*, *FUBP1*, *NDUFS3*, *RAPSN*	Associated with increased BMI
Wang et al., 2011 [80]	Non-Hispanic Caucasians	*FTO*, *NRXN3*	*FTO* associated with increased BMI and *NRXN3* with higher WHR
Yengo et al., 2018 [39]	European ancestry	*HSD17B12*, *STAG3L1*, *CAMKV*	Associated with decreased adiposity

**Table 3:** Summary of epigenome-wide association studies (EWAS) identifying DNA methylation signatures associated with obesity-related traits.

**Study ID**	**Study Population**	**Key Gene(s)/Locus (Selected)**	**Key Findings**
Taylor et al., 2023 [81]	Black/African American	*SOCS3*, *RALGDS*, *PSKH1*, *FGD2*, *BMP6*, *TSLP*	Hypomethylation in these genes was linked to higher BMI
Do et al., 2023 [82]	Multi-ethnic	*TOP1MT*, *TNFRSF13B*, *LGALS3BP*	Hypermethylation at *TOP1MT* was positively associated and another two were negatively associated with BMI
Alfano et al., 2023 [83]	European population	*ARID5B*, *KLF9*, *PCSK5*	Methylation in these genes positively associated with rapid weight growth
Ali et al., 2016 [84]	Northern European ancestry	*SOCS3*	Hypomethylation of *SOCS3* associated with higher risk of obesity
Aslibekyan et al., 2015 [51]	European American	*CPT1A*, *PHGDH CD38*, *LINC00263*	Hypermethylation at *CPT1A* and *PHGDH* associated with lower adiposity and *CD38* and *LINC00263* associated with increased adiposity
Campanella et al., 2018 [52]	European ancestry	*ABCG1*	Methylation at the CpG site in the gene *ABCG1* showed association with BMI, WC, WHR, and WHtR
Chen et al., 2021 [85]	Multi-ethnic Asian individuals	*THADA*, *TNIK*, *RSRC1*, *ETAA1*	Methylation near *THADA* and *TNIK* is linked to lower BMI, while *RSRC1* and *ETAA1* are linked to higher BMI and WC
Demerath et al., 2015 [48]	African Americans	*ABCG1*, *SREBF1*, *KDM2B*, *CPT1A*, *LGALS3BP*, *PBX1*, *BBS2*, *DHCR24*	Methylation near *ABCG1*, *SREBF1*, *KDM2B*, *LGALS3BP*, *PBX1*, and *BBS2* was positively associated with BMI and/or WC, while *CPT1A* and *DHCR24* showed negative associations
Dhana et al., 2018 [86]	European and African American	*MSI2*, *LARS2*, *ABCG1*, *SREBF1*, *LGALS3BP*, *BRDT CPT1A*, *TMEM49*	Methylation at *MSI2*, *LARS2*, *ABCG1*, *SREBF1*, and *LGALS3BP* (positive) and *CPT1A*, *TMEM49*, and *BRDT* (negative) associated with BMI and/or WC
Kvaløy et al., 2018 [87]	Norwegian women	*RPS6KA2*, *DMAP1*, *SETBP1*	Methylation at *RPS6KA2*, *DMAP1*, and *SETBP1* negatively associated with BMI
Lee et al., 2021 [53]	European ancestry	*CPT1A*, *ABCG1*	Methylation in CPT1A and ABCG1 associated with BMI
Li et al., 2024 [88]	Han Chinese	*TRIM15*, *SLC38A4*	Hypermethylation at *SLC38A4* associated with a decrease in BMI and *TRIM15* associated with increase in BMI
Meeks et al., 2017 [55]	Sub-Saharan African	*CPT1A*	Hypomethylation of *CPT1A* associated with increased BMI, obesity, WC, and abdominal obesity
Nikpay et al., 2021 [89]	Primarily European ancestry	*CCNL1*, *SLC5A11*, *MAST3*, *POMC*, *ADCY3*, *DNAJC27*	Hypomethylation at *CCNL1* and *SLC5A11* and hypermethylation at *MAST3*, *POMC*, *ADCY3*, and *DNAJC27* associated with increased BMI
Sayols-Baixeras et al., 2017 [90]	European ancestry	*SREBF1*, *NOTCH4 SLC7A11*, *CPT1A*, *SYNGAP1*, *GRIK1*, *CACNA1C*, *CUX1*	Hypermethylation of *CUX1*, *SREBF1*, *SLC7A11*, *SYNGAP1*, and *GRIK1* and hypomethylation of *CPT1A*, *CACNA1C*, and *NOTCH4* are linked to higher BMI and/or WC
Vehmeijer et al., 2020 [91]	Primarily European ancestry	*SFRP5*, *SLC43A2*, *SFXN5*	Hypermethylation at CpGs in *SFRP5*, *SLC43A2*, and *SFXN5* was positively associated with increased BMI
Wahl et al., 2017 [54]	European and Indian Asian	*ABCG1*, *SREBF1*, *SOCS3*, *CPT1A*	Hypermethylation at *ABCG1*, *SREBF1*, and *SOCS3* and hypomethylation at *CPT1A* associated with increased BMI
Wang et al., 2018 [92]	African American	*SBNO2*, *SOCS3*, *CISH*, *PIM3*, *KLF4*	Hypermethylation at *SBNO2* and hypomethylation at *SOCS3*, *CISH*, *PIM3*, and *KLF4* associated with higher obesity
Xie et al., 2021 [93]	European ancestry	*ST8SIA5*	Hypermethylation near *ST8SIA5* associated with higher WC
Zhao et al., 2023 [94]	Chinese ancestry	*RPS6KA2*, *RPTOR*, *ZNF827*, *KSR1*, *NFIC*	Hypomethylation at *RPS6KA2*, *RPTOR*, and *ZNF827* and hypermethylation at *KSR1* and *NFIC* are linked to higher BMI and WHR

## Data Availability

This is a review article, and all data supporting the findings of this study are available within the manuscript. No additional data were generated or analyzed.

## Abbreviations

The following abbreviations are used in this manuscript:

***A****BCG1*, ATP Binding Cassette Subfamily G Member 1; *ADAM23*, ADAM Metallopeptidase Domain 23; *ADAMTS*, ADAM Metallopeptidase with Thrombospondin Type 1 Motif; *ADCY3*, Adenylate Cyclase 3; AOM, Azoxymethane; *AP2K5*, AP2 Associated Kinase 5; *APOBR*, Apolipoprotein B Receptor; *APOE*, Apolipoprotein E; *ARID5B*, AT-Rich Interaction Domain 5B; ***B****BS2*, Bardet–Biedl Syndrome 2; BCAA, branched-chain amino acid; *BDNF*, Brain-Derived Neurotrophic Factor; BMI, Body Mass Index; *BMP6*, Bone Morphogenetic Protein 6; *BRDT*, Bromodomain Testis-Specific; ***C****ACNA1C*, Calcium Voltage-Gated Channel Subunit Alpha1 C; *CAMKV*, CaM Kinase V; *CCDC92*, Coiled-Coil Domain Containing 92; *CCNL1*, Cyclin L1; *CCNT2*, Cyclin T2; *CD38*, Cluster of Differentiation 38; *CHRNB4*, Cholinergic Receptor Nicotinic Beta 4 Subunit; *CISH*, Cytokine Inducible SH2 Containing Protein; CNS, central nervous system; *CPEB4*, Cytoplasmic Polyadenylation Element Binding Protein 4; CpG, Cytosine-phosphate-Guanine; *CPT1A*, Carnitine Palmitoyltransferase 1A; *CTNNBL1*, Catenin Beta Like 1; *CTSS*, Cathepsin S; *CUX1*, Cut-Like Homeobox 1; ***D****HCR24*, 24-Dehydrocholesterol Reductase; *DMAP1*, DNA Methyltransferase 1 Associated Protein 1; DNA, Deoxyribonucleic Acid; *DNAJC27*, DnaJ Heat Shock Protein Family (Hsp40) Member C27; *DNM1*, Dynamin 1; ***E****LP3*, Elongator Acetyltransferase Complex Subunit 3; *ENPP1*, Ectonucleotide Pyrophosphatase/Phosphodiesterase 1; *EP300*, E1A Binding Protein P300; *ETAA1*, E1A Associated Protein 1; EWAS, Epigenome-Wide Association Study; ***F****ADS1*, Fatty Acid Desaturase 1; *FADS2*, Fatty Acid Desaturase 2; *FAIM2*, Fas Apoptotic Inhibitory Molecule 2; *FGD2*, FYVE, RhoGEF, and PH Domain Containing 2; *FTO*, Fat Mass And Obesity Associated; *FUBP1*, Far Upstream Element Binding Protein 1; ***G****CKR*, Glucokinase Regulator; GIANT, Genetic Investigation of Anthropometric Traits; GLP-1, Glucagon-Like Peptide-1; *GNPDA2*, Glucosamine-6-Phosphate Deaminase 2; *GPR101*, G Protein-Coupled Receptor 101; *GPR75*, G Protein-Coupled Receptor 75; *GRB14*, Growth Factor Receptor Bound Protein 14; *GRIK1*, Glutamate Ionotropic Receptor Kainate Type Subunit 1; GRS, genetic risk score; GWAS, Genome-Wide Association Study; ***H****IF3A*, Hypoxia Inducible Factor 3 Subunit Alpha; *HOXC13*, Homeobox C13; *HSD17B12*, Hydroxysteroid 17-Beta Dehydrogenase 12; ***I****NPP4B*, Inositol Polyphosphate-4-Phosphatase B; *IRS1*, Insulin Receptor Substrate 1; *IRX3*, Iroquois Homeobox 3; *IRX5*, Iroquois Homeobox 5; *ITPR2*, Inositol 1,4,5-Trisphosphate Receptor Type 2; ***K****CNQ1*, Potassium Voltage-Gated Channel Subfamily Q Member 1; *KCTD15*, Potassium Channel Tetramerization Domain Containing 15; *KDM2B*, Lysine Demethylase 2B; *KLF4*, Kruppel-Like Factor 4; *KLF9*, Kruppel-Like Factor 9; *KSR1*, Kinase Suppressor Of Ras 1; ***L****ARS2*, Leucyl-tRNA Synthetase 2, Mitochondrial; *LEKR1*, Leukocyte Receptor Transmembrane Protein 1; *LEP*, Leptin; *LEPR*, leptin receptor; *LGALS3BP*, Galectin 3 Binding Protein; *LINC00263*, Long Intergenic Non-Protein Coding RNA 263; *LYPLAL1*, Lysophospholipase Like 1; ***M****AP2K5*, Mitogen-Activated Protein Kinase 5; *MAST3*, Microtubule Associated Serine/Threonine Kinase 3; *MC4R*, Melanocortin 4 Receptor; miRNA, microRNA; *MSI2*, Musashi RNA Binding Protein 2; *MTOR*, Mechanistic Target Of Rapamycin Kinase; ***N****DUFS3*, NADH: Ubiquinone Oxidoreductase Core Subunit S3; NEGR, Not Elsewhere Given; *NEGR1*, Neuronal Growth Regulator 1; *NFIC*, Nuclear Factor I C; NIH, National Institutes of Health; *NLK*, Nemo Like Kinase; *NOTCH4*, Notch Receptor 4; *NRXN3*, Neurexin 3; ***P****BX1*, Pre-B Cell Leukemia Homeobox 1; *PCSK1*, Proprotein Convertase Subtilisin/Kexin Type 1; *PCSK5*, Proprotein Convertase Subtilisin/Kexin Type 5; *PEX2*, Peroxisomal Biogenesis Factor 2; *PGC1A*, PPARG Coactivator 1 Alpha; *PHGDH*, Phosphoglycerate Dehydrogenase; *PIM3*, Proviral Integration Site Moloney Murine Leukemia Virus 3; *POMC*, proopiomelanocortin; *PPARG*, Peroxisome Proliferator Activated Receptor Gamma; PRS, polygenic risk score; *PSKH1*, Protein Serine/Threonine Kinase H1; ***R****AB27B*, RAB27B, Member RAS Oncogene Family; *RALGDS*, Ral Guanine Nucleotide Dissociation Stimulator; *RAPSN*, Receptor Associated Protein Of The Synapse; *RBFOX1*, RNA Binding Fox-1 Homolog 1; RNA, Ribonucleic Acid; *RPS6KA2*, Ribosomal Protein S6 Kinase A2; *RPTOR*, Regulatory Associated Protein With MTOR Complex 1; *RSPO3*, R-Spondin 3; *RSRC1*, Arginine And Serine Rich Coiled-Coil 1; ***S****BNO2*, Strawberry Notch Homolog 2; SCFA, short-chain fatty acid; *SEC16B*, SEC16 Homolog B, SEC16 homolog B, Endoplasmic Reticulum Export Factor; *SETBP1*, SET Binding Protein 1; *SFRP5*, Secreted Frizzled Related Protein 5; *SFXN5*, Sideroflexin 5; *SLC32A1*, Solute Carrier Family 32 Member 1; *SLC38A4*, Solute Carrier Family 38 Member 4; *SLC43A2*, Solute Carrier Family 43 Member 2; *SLC5A11*, Solute Carrier Family 5 Member 11; *SLC7A11*, Solute Carrier Family 7 Member 11; SNP, Single Nucleotide Polymorphism; *SOCS3*, Suppressor Of Cytokine Signaling 3; SPI, Spirometry; *SREBF1*, Sterol Regulatory Element Binding Transcription Factor 1; *SSFA2*, Sperm-specific antigen 2; *ST8SIA5*, ST8 Alpha-Sialidases 5; *STAG3L1*, Stromal Antigen 3 Like 1; *STX4*, Syntaxin 4; *SYNGAP1*, Synaptic Ras GTPase Activating Protein 1; ***T****AS2R38*, Taste 2 Receptor Member 38; *TCF7L2*, Transcription Factor 7 Like 2; *THADA*, Thyroid Adenoma-Associated Protein; *TMEM18*, Transmembrane Protein 18; *TMEM49*, Transmembrane Protein 49; *TNFRSF13B*, TNF Receptor Superfamily Member 13B; *TNIK*, TRAF2 And NCK Interacting Kinase; *TOP1MT*, Topoisomerase I, Mitochondrial; *TRIM15*, Tripartite Motif Containing 15; *TSLP*, Thymic Stromal Lymphopoietin; *TUFM*, Tu translation elongation factor, mitochondrial; *TXNDC12*, Thioredoxin Domain Containing 12; ***V****EGFA*, Vascular Endothelial Growth Factor A; **W**C, waist circumference; WHO, World Health Organization; WHR, Waist–Hip Ratio; *ZNF827*, Zinc Finger Protein 827.
